# *C. elegans*—An Emerging Model to Study Metal-Induced RAGE-Related Pathologies

**DOI:** 10.3390/ijerph15071407

**Published:** 2018-07-04

**Authors:** Adi Pinkas, Airton Cunha Martins, Michael Aschner

**Affiliations:** Albert Einstein College of Medicine, Jack and Pearl Resnick Campus, 1300 Morris Park Avenue, Forchheimer Building, Room 209, Bronx, New York, NY 10461, USA; airtoncmjr@gmail.com (A.C.M.J.); michael.aschner@einstein.yu.edu (M.A.)

**Keywords:** receptor for advanced glycation end products, methylmercury, selenium, zinc, arsenic, manganese, *C. elegans*

## Abstract

The receptor for advanced glycation end products (RAGE), a multi-ligand receptor, is mostly associated with promoting inflammation and oxidative stress. In addition to advanced glycation end products (AGEs), its ligands include High mobility group box 1 protein (HMGB-1), S-100 proteins and beta-sheet fibrils. The effects of several metals and metalloids on RAGE expression and activation have been recently studied: in vivo and in vitro exposure to methylmercury, selenium, zinc, manganese, and arsenic was associated with a variety of RAGE-related alterations and behavioral impairments, which are mostly dependent upon the administration procedure (local vs. systemic) and age during exposure. Recently, *C. elegans* has been proposed as a potential novel model for studying RAGE-related pathologies; preliminary data regarding such model and its potential contribution to the study of metal-induced RAGE-related pathologies are discussed.

## 1. Introduction

The receptor for advanced glycation end products (RAGE) is a mammalian, multiligand receptor of the immunoglobulin family. RAGE is found on the surface of a variety of cell types and was first identified and described in 1992, initially as a specific binding site for advanced glycation end products (AGEs) [1]. RAGE is expressed in different cell types such as neurons, dendritic cells, neutrophils, monocytes/macrophages, lymphocytes, cardiomyocytes and vascular endothelial [2].

RAGE can bind to different ligands such as AGEs, Aβ, fibrillar Aβ, high-mobility group box protein (HMGB1), macrophage-antigen-1 (Mac1), S-100 proteins, β-amyloid, β-sheet fibrils, and lipopolysaccharide [3,4,5,6]. This binding of RAGE and its ligands results in depletion of cellular antioxidant defense mechanisms and generation of reactive oxygen species. It also induces up-regulation of inflammatory cytokines through RAGE signal transduction and activation of transcription factors that are associated with pathologies, such as diabetes, atherosclerosis, coronary artery disease, cancer, and neurodegenerative diseases [6,7,8,9,10].

Previous reports indicate that chemical elements can affect RAGE expression and activation and lead to changes in cell signaling pathways which, in turn, are associated with the onset and exacerbation of diabetes, hypertension, cancer and also neurodegenerative illnesses such as Alzheimer’s disease (AD) and Parkinson’s disease [11,12,13]. Several chemical elements (metals, metalloids, and non-metals) play an essential role in human life and are considered/assumed to be essential to metabolic function. However, some essential elements such as selenium and manganese can be recognized as toxic in excess [14]. On the other hand, some elements are considered “toxic elements”, denoting that even at low levels of exposure some adverse effect is likely to occur; such is the case for arsenic or mercury exposure [14].

In this context, the present review focuses on the role of RAGE in neurodegenerative and other diseases which are related to exposure to specific elements (Table S1). In addition, the potential contribution of a novel *C. elegans* model for the study of RAGE-related metal-induced neurodegeneration is discussed.

## 2. MeHg

Mercury is a heavy metal, which can be found in different chemical forms: elemental or Hg^0^, inorganic Hg compounds (Hg^+^ or Hg^2+^) and organic compounds, such as ethylmercury (EtHg) and methylmercury (MeHg) [15,16]. Excessive exposure to MeHg occurs, most commonly, through consumption of contaminated fish and seafood. Several studies highlight the ability of MeHg to cross the blood-brain barrier (BBB) and cause neurotoxicity and it can lead to impairments in developing brains in fetuses and children [12,17,18].

In a recent study, developmental neurotoxicity following prenatal exposure to MeHg was studied in rats: pregnant rats were exposed by gavage between gestational day 5 and parturition and the offspring were tested behaviorally and their brains were analyzed [12]. Using an open-field test, decreased exploration behavior and increased anxiety-like behavior were observed in rats prenatally exposed to MeHg; the novel-object recognition task unveiled a deficit in short and long-term memory in these rats as well. Along with these behavioral findings, hippocampal decrease in RAGE expression was found in brains of MeHg exposed rats.

To better understand the role of mercury exposure in Alzheimer’s disease (AD) commencement and exacerbation, another study focused on the effects of MeHg exposure on amyloid β-protein (Aβ) in the brains of adult rats [19]. Following chronic administration by gavage, hippocampal levels of Aβ increased in a dose-dependent manner while levels of RAGE increased in the brain capillary endothelium. As RAGE plays a crucial role in transferring Aβ from the interstitial fluid into the brain [20,21], it is argued that the detrimental effects of MeHg may not be related to generation and degradation of Aβ in the brain, but more likely to Aβ transport to and from the brain.

## 3. Selenium

Selenium is a nonmetal found in rocks, soil, and food; fruits, vegetables, and animal protein are the main sources of the element. Selenium is involved in several biological pathways as an integral component of selenoproteins such as glutathione peroxidase, an enzyme that scavenges for ROS and plays an important role in oxidative injury prevention [22,23]. Moreover, selenium regulates the activity of various enzymes involved in the processes of glycolysis and gluconeogenesis [24,25]. Low selenium intake is associated with developmental diseases and overexposure to selenium results in toxicity [26].

Among the many processes affected by selenium are inflammation, oxidative stress, hormone production and DNA methylation and repair [27]. Furthermore, selenium exhibits several insulin-like effects related to glucose transport and glycolysis [28,29]. In an effort to conduct an in-depth study of the insulin-like effects of selenium, a streptozotocin-induced rat model for diabetes was used: in addition to mitigating diabetes-related altered activity of antioxidant enzymes and glycated hemoglobin content, chronic selenium administration also lead to downregulation of nuclear factor kappa-light-chain-enhancer of activated B cells (NF-kB) and RAGE expression [30]. This effect on RAGE was observed in both control and diabetic animals and the offered explanation was decreased levels of glycosylated hemoglobin, secondary to the hypoglycemic effect of selenium. Finally, it was hypothesized that selenium’s effect on RAGE and NF-kB would also lead to a reduction in diabetes-related inflammation; this was, indeed, confirmed by histopathological studies.

## 4. Zinc and Manganese

Zinc (Zn) is an essential element that is a component of several enzymes and is very important to biochemical and physiological processes related to glucose metabolism, as it participates in the synthesis, storage, secretion, and translocation of insulin [31,32,33]. Several epidemiologic studies have reported a correlation between low levels of zinc and the occurrence of type 2 diabetes mellitus [34,35,36,37,38]. Zinc also plays a vital role in the maintenance and regulation of the immune system [39].

Manganese (Mn) is an essential dietary nutrient found in many foods such as legumes, grains, nuts, rice, beans, and tea [40]. The metal is required for normal development and growth, as it regulates bone formation, the immune and reproduction systems, and protein, carbohydrate and lipid metabolism. Furthermore, manganese is used as a cofactor for several enzymes such as glutamine synthetase, arginase and superoxide dismutase [41]. However, chronic exposure to high levels of manganese may lead to toxicity, a condition referred to as manganism which results in extrapyramidal motor disorders [42,43]. In addition, such exposure results in its accumulation in some brain regions—a known risk factor for Parkinson’s disease [44,45].

The effects of zinc and manganese on AGE-mediated endothelial cell dysfunction were studied in vitro using bovine aortic endothelial cells (BAECs) [13]. Initial exposure of the cells to AGEs was followed by treatment with either zinc or manganese: when compared to cells which went through AGEs exposure and no treatment, treated cells exhibited increased cell viability in both cases; additionally, zinc treatment lead to a significant decrease in NF-kB activation and RAGE expression and manganese treatment lead to significant downregulation of NF-kB expression and nuclear translocation. A sizable nonsignificant decrease in RAGE expression was also observed following manganese treatment, possibly due to the small number of subjects (N = 3). Future studies should focus on the effects of these two metals on RAGE, since both metals showed a similar overall tendency, namely decreased RAGE expression and, perhaps subsequently, mitigated inflammation.

## 5. Arsenic

Arsenic (As) is a metalloid that is widely distributed throughout the Earth’s crust in a variety of chemical forms, including organic and inorganic species [46]. The inorganic arsenic species are known human carcinogens, being found mainly in drinking water and foods such as rice, fish, and vegetables [47,48]. The neurotoxic effects of arsenic exposure have been previously discussed: while childhood exposure to arsenic may lead to reduced intellectual function and cognitive abilities, later exposure may result in encephalopathies, memory, learning and concentration decline, and mood disorders such as depression and anxiety [49,50,51].

Arsenic exposure has been associated with several peripheral vascular disease, diabetes, chronic lung disease and also several types of cancer [52,53,54,55,56]. As most studies focus on high levels of exposures, Lantz et al. studied the effects of low-dose chronic arsenic exposure on pulmonary protein expression [57]. In their study, levels of several proteins in the lung-lining fluid were evaluated in adult mice following a 4-week exposure to arsenic in drinking water: a negative correlation between arsenic exposure dosage and expression of RAGE, with a higher dose leading to a larger decrease in expression. To test this correlation in a human context, levels of RAGE expression from sputum samples were compared with urinary arsenic concentration from the same subjects: here too a negative correlation was found, as subjects with higher urinary arsenic concentrations had lower levels of RAGE expression. Two suggested mechanisms for these findings are arsenic-induced alteration of promoter region methylation and also altered transcriptional regulation of RAGE.

Chronic arsenic exposure has also been associated with several neurodegenerative phenomena, including demyelination, altered chemical transmission, DNA defragmentation and lipid peroxidation; in many cases, these processes are secondary to increased generation of reactive species. It has recently been hypothesized that the deleterious effects arsenic exposure has on the nervous system, over time, lead to the commencement and exacerbation of Alzheimer’s disease (AD) via Aβ production and related pathways. This notion was recently addressed using the rat model, in a study where animals were exposed chronically to arsenic beginning fetal development and ending at 4 months of age; following exposure, the animals were tested behaviorally and their brains were subsequently analyzed [11]. Using a contextual fear conditioning test, arsenic exposure was associated with behavioral impairments; complimentary to this observation, levels of both RAGE and Aβ were elevated in the brains of exposed animals. Finally, levels of the low-density lipoprotein receptor-related protein 1 (LRP1) were also evaluated, as this protein mediates Aβ clearance by transferring it from the brain to the bloodstream [58]: levels of this protein were unchanged following arsenic exposure which, along with elevated RAGE, further confirms the theory of arsenic-related generation and accumulation of Aβ.

## 6. *C. elegans* Model for RAGE Study

*Caenorhabditis elegans* (*C. elegans*) is an expedient experimental worm-model due to its characteristics, namely small size, short lifespan, rapid life cycle and translucent body, to name a few [59,60,61]. Most of the *C. elegans* genes and almost half of its disease-related genes are evolutionarily conserved and this worm has been used to study numerous human diseases and neurodegenerative disorders, such as AD, Parkinson’s disease (PD), stroke, cancer and metabolic diseases [62,63,64]. Recently, the potential benefits of using the *C. elegans* model for studying RAGE-related pathology have been discussed, as very few mammals (in vivo) and cell lines (in vitro) are currently used in this context [3,65]. For example, the effects of RAGE expression on *daf-16* pathways could be further investigated in the worm, as these pathways seem to mediate the ameliorative effects of human insulin on lifespan reduction and neuronal damage—two phenomena which are exhibited following exposure to high glucose conditions [66].

Subsequently, a *C. elegans* strain expressing RAGE in neurons was generated to serve as a model for studying RAGE-related pathologies [67]. Initial characterization of this worm included behavioral, developmental and morphological assessment. Locomotion was evaluated in this strain in three different contexts: general locomotion (no treatment), locomotion following heat shock and locomotion following ethanol exposure. A decrease in general locomotion was observed using a method of counting body bends (less body bends per minute for RAGE worms). Following heat shock and ethanol exposure, a normal decrease in locomotion is observed in the wild-type (N2); the RAGE worm, however, exhibited a significantly larger decrease in locomotion following these exposures. Another behavior which was tested in the RAGE worm is pharyngeal pumping following heat-shock: here too, N2 shows a decrease in pharyngeal pumping following such exposure. Indeed, the RAGE worm exhibits a larger decrease in locomotion following heat-shock. Regarding development, two parameters were evaluated in the RAGE worm as compared with N2: (1) the percentage of hatched worms 24 h after egg laying and (2) the time it takes an egg to develop into an egg-laying adult. An overall developmental delay was found, with a significant decrease of hatched RAGE-laid eggs after 24 h and also a longer amount of time needed for RAGE worms to reach the egg-laying stage during adulthood. Finally, the RAGE worm strain was crossed with several other strains, which have different neurotransmitter systems tagged with GFP (dopaminergic, serotonergic, cholinergic, GABAergic and glutamatergic). Using confocal microscopy, fluorescent signaling was evaluated in these crossed worms to study RAGE-related neurodegeneration; a decrease in fluorescent signaling was found only in worms with GFP-tagged dopaminergic system, suggesting some impairments to that system specifically.

In an extensive review regarding the use of *C. elegans* as a platform for studying metals-related neurodegenerative diseases, the role of aluminum (Al), copper (Cu), iron (Fe), lead (Pb), manganese (Mn), mercury (Hg), and zinc (Zn) in AD, PD, and other neurodegenerative diseases is discussed: indeed, *C. elegans* proved to be a highly efficient screening system for metal-induced neurodegeneration, as the use of this model lead to an in-depth understanding of metal transportation and homeostasis, along with an insight into potential therapeutic methods for neurodegenerative diseases [68].

Considering this tremendous potency of the *C. elegans* model, the recently developed RAGE-expressing worm strain is suggested as a favorable candidate for elucidating the role of RAGE in metal-induced neurodegeneration. The ease and speed associated with this model, together with its conserved neurotransmitter biology, provide the ideal scaffolding for building a more efficient experimental design and, ultimately, should greatly contribute to the study of RAGE-related metal neurotoxicity and the development of relevant and efficient therapeutic strategies.

## Supplementary Materials

The following are available online at http://www.mdpi.com/1660-4601/15/7/1407/s1, Table S1: The effects of metal toxicity on RAGE expression.
