# Supplementary material for: C. elegans—An Emerging Model to Study Metal-Induced RAGE-Related Pathologies
Source: Int J Environ Res Public Health. 2018 Jul 4;15(7):1407. doi: 10.3390/ijerph15071407 (PMC6069300; doi:10.3390/ijerph15071407)
Supplement: Supplementary file 1 [file ijerph-15-01407-s001.pdf]

Table S1. The effects of metal toxicity on RAGE expression.

Metal	Model	Exposure	Behavior	Molecular	Reference
MeHg	Rat	Prenatal	decreased exploration behavior; increased anxiety-like behavior; impaired short and long-term memory	hippocampal decrease in RAGE expression	[1]
MeHg	Rat	Chronic		increased levels of hippocampal Aβ; increased levels of RAGE in the brain capillary endothelium	[2]
Selenium	Rat model of diabetes	Chronic		downregulation of NF-kB and RAGE expression; reduced diabetes-related inflammation	[3]
Zinc	Bovine aortic endothelial cells	Exposure to AGEs followed by treatment with zinc		significant decrease in NF-kB activation and RAGE expression	[4]
Manganes e	Bovine aortic endothelial cells	Exposure to AGEs followed by treatment with manganese		downregulation of NF-kB expression and nuclear translocation	[4]
Arsenic	Mice	Chronic		decrease in RAGE expression	[5]
Arsenic	Human	None; sample evaluation		higher urinary arsenic concentrations correlated with lower levels of RAGE expression in sputum samples	[5]

1. Heimfarth, L.; Delgado, J.; Mignori, M.R.; Gelain, D.P.; Moreira, J.C.F.; Pessoa-Pureur, R. Developmental neurotoxicity of the hippocampus following in utero exposure to methylmercury: Impairment in cell signaling. *Arch. Toxicol.* **2018**, *92*, 513–527.
2. Kim, D.K.; Park, J.D.; Choi, B.S. Mercury-induced amyloid-beta (abeta) accumulation in the brain is mediated by disruption of abeta transport. *J. Toxicol. Sci.* **2014**, *39*, 625–635.
3. Pillai, S.S.; Sugathan, J.K.; Indira, M. Selenium downregulates rage and nfkappab expression in diabetic rats. *Biol. Trace Elem. Res.* **2012**, *149*, 71–77.
4. Zhuang, X.; Pang, X.; Zhang, W.; Wu, W.; Zhao, J.; Yang, H.; Qu, W. Effects of zinc and manganese on advanced glycation end products (ages) formation and ages-mediated endothelial cell dysfunction. *Life Sci.* **2012**, *90*, 131–139.
5. Lantz, R.C.; Lynch, B.J.; Boitano, S.; Poplin, G.S.; Littau, S.; Tsaprailis, G.; Burgess, J.L. Pulmonary biomarkers based on alterations in protein expression after exposure to arsenic. *Environ. Health Perspect.* **2007**, *115*, 586–591.
